# Clinical and Demographic Characteristics of Patients Hospitalized for Decompensated Heart Failure with Extremely High NT-proBNP Levels

**DOI:** 10.3390/diagnostics14222507

**Published:** 2024-11-09

**Authors:** Ruxandra Maria Christodorescu, Daniel Miron Brie, Alina Diduța Brie, Samuel Nistor, Alexandru Tîrziu, Angela Dragomir, Cristian Mornoș, Simona Drăgan, Daniel Duda-Seiman, Adina Pop-Moldovan, Dan Dărăbanțiu

**Affiliations:** 1 Department of Internal Medicine, “Victor Babes” University of Medicine and Pharmacy, Eftimie Murgu Square, No. 2, 300041 Timisoara, Romania; 2 Research Center of the Institute of Cardiovascular Diseases, Cardiovascular Disease Institute Timisoara, Gheorghe Adam St., No. 13A, 300310 Timisoara, Romania; ange.dragomir@gmail.com (A.D.); simona.dragan@umft.ro (S.D.); daniel.duda-seiman@umft.ro (D.D.-S.); 3 Cardiovascular Disease Institute Timisoara, Gheorghe Adam St., No. 13A, 300310 Timisoara, Romania; nistor_samuel@yahoo.com (S.N.); alexandru.tirziu@umft.ro (A.T.); 4 Department of Cell and Molecular Biology, “Victor Babes” University of Medicine and Pharmacy, Tudor Vladimirescu Street, No. 14, 300174 Timisoara, Romania; 5 ANAPATMOL Research Center, “Victor Babes” University of Medicine and Pharmacy, Tudor Vladimirescu Street, No. 14, 300174 Timisoara, Romania; 6 Center of Modeling Biological Systems and Data Analysis, “Victor Babes” University of Medicine and Pharmacy, 300174 Timisoara, Romania; 7 Department of Functional Sciences, “Victor Babes” University of Medicine and Pharmacy, Tudor Vladimirescu Street, No. 14, 300174 Timisoara, Romania; 8 Department of Cardiology, “Victor Babes” University of Medicine and Pharmacy, Eftimie Murgu Square, No. 2, 300041 Timisoara, Romania; 9 Faculty of Medicine, “Vasile Goldis” Western University of Arad, Revolutiei Bvd., No. 174, 310025 Arad, Romania; ppmldvn68@yahoo.com; 10 Arad County Emergency Clinical Hospital, 310158 Arad, Romania; ddarabantiu@yahoo.com

**Keywords:** heart failure, biomarkers, NT-proBNP level, risk factors

## Abstract

Background: NT-proBNP levels with a wide range at admission play both a diagnostic and a prognostic role in patients with HF. The differences regarding the clinical profiles and demography in decompensated HF patients according to NT-proBNP levels at admission are not clear. Methods: This study aimed to analyze and compare clinical profiles and demographics in patients hospitalized for decompensated heart failure according to levels of NT-proBNP at admission. The study included 302 patients hospitalized for decompensated HF who were divided into three groups based on admission NT-proBNP levels: group A (n = 46, with NT-proBNP level < 3000 pg/mL), group B (n = 130, NT-proBNP level between 3000–10,000 pg/mL), and group C (n = 126, NT-proBNP level > 10,000 pg/mL). Results: Patients hospitalized with decompensated HF and very high levels of NTproBNP, above 10,000 pg/mL at admission, are older, have a lower LVEF, higher NYHA class, more renal dysfunction, and longer hospital stay, resulting in a more severe clinical profile. Conclusions: The presence of very high levels of NT-proBNP may identify a category of patients with a more severe prognosis that requires more aggressive management and closer follow-up.

## 1. Introduction

Heart failure (HF) is a syndrome that is characterized by reduced functional capacity, repeated hospitalizations, poor quality of life, and high mortality [1]. The prevalence of heart failure is estimated at 64 million patients worldwide [2], with 17 cases (inter-quartile range, IQR, 14–21) per 1000 patients in Europe [3], but with high variability between countries [3,4].

The prevalence of HF is relatively high in Central European countries [5], being 4.7% of patients over 35 years of age in Romania [6]. 

According to the ESC Guidelines published in 2021, HF is classified based on the LV ejection fraction in HF with reduced EF (HFrEF, LVEF ≤ 40%), mildly reduced EF (HFmrEF, LVEF between 41–49%), and preserved EF (HFpEF, LVEF ≥ 50%) [7].

Based on the data from the ESC Long-Term Registry [8,9], 60% of patients presented heart failure with reduced ejection fraction (HFrEF, LVEF < 40%), 24% with moderately reduced ejection fraction (HFmrEF, LVEF 40–49%), and 16% with preserved ejection fraction (HFpEF, LVEF > 50%) [10].

Approximately 60–80% of patients with decompensated heart failure are initially diagnosed in the emergency department [11].

Decompensated HF (worsening HF) is defined as the onset or worsening of signs and/or symptoms of HF that require initiation or intensification of diuretic therapy [12]. N-terminal pro-B-type natriuretic peptide (NT-proBNP) is a useful biomarker in the diagnosis [13,14,15] and prognosis of HF [16], as it is the most utilized peptide for diagnosing and managing heart failure in Europe [9]. Although both BNP and NT-proBNP may be used for ruling in HF, their half-lives differ. The estimated half-life for BNP is ∼21 min, whereas for NT-proBNP it is extended to around 70 min. Consequently, concentrations of NT-proBNP are higher than those of BNP [17]. Because of its stability at room temperature, this marker is easier to measure in most hospital laboratories. Additionally, there is no need for correction regarding gender, BMI, kidney function, and LVEF. All these characteristics make NT-proBNP a more convenient molecule to work with than BNP [17]. Levels of NT-proBNP may vary significantly across all ranges of EF in patients with decompensated HF. However, the significance of highly elevated levels of this biomarker above 10,000 pg/mL at admission in patients with HF and its prognostic importance is unclear.

New literature data suggest that NT-proBNP is a powerful marker for ruling in/out the diagnosis of heart failure. Using NT-proBNP for guiding medical therapy has resulted in reductions in initial hospitalizations, admission to cardiology departments and intensive care units, and hospital readmission, leading to a lower inpatient management costs [11].

This study analyzed the clinical, biological, and demographic data at hospital admission of in patients with decompensated heart failure (worsening HF) who were referred to a county hospital in the western part of Romania. Among patients with elevated levels of NT-proBNP admitted for decompensated HF, a subgroup with very high levels (>10,000 pg/mL) was identified and analyzed. 

## 2. Materials and Methods

**Figure 1:**
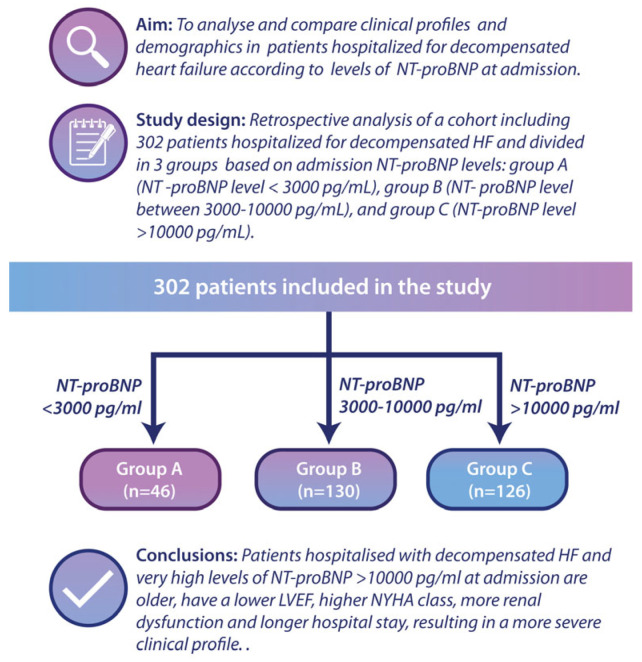
Graphical abstract presenting the study design. A total of 302 patients were divided into three groups: Group A—NT-proBNP < 3000 pg/mL; Group B—NT-proBNP between 3000–10,000 pg/mL; Group C—NT-proBNP > 10,000 pg/mL.

The study design is presented in the graphical abstract (Figure 1).

The aim of this study is to analyse and compare the clinical characteristics and demographics of patients admitted for decompensated heart failure, categorized by their NT-proBNP levels at the time of admission. This retrospective study included 302 consecutive patients hospitalized for decompensated HF (defined as the presence of signs of pulmonary and/or systemic congestion that required at least 40 mg of intravenous furosemide and elevated NT-proBNP levels). The following inclusion criteria were considered: adults (>18 years old) who signed the informed consent form and who presented at the emergency department of a county hospital with signs and symptoms of decompensated heart failure. The exclusion criteria were female patients with pregnancy or breastfeeding, those refusing to give informed consent, those with acute coronary syndrome requiring emergency coronary angiography, and patients with aortic dissection, pulmonary embolism, or those with pulmonary arterial hypertension from causes other than left ventricular dysfunction. The patients were divided at admission into three groups according to the NT-proBNP level: group A (n = 46, with NT-proBNP level < 3000 pg/mL), group B (n = 130, NT-proBNP level between 3000–10,000 pg/mL), and group C (n = 126, NT-proBNP level > 10,000 pg/mL). 

All patients had ECG, echocardiography, and biological tests performed on admission. In addition to the standard biological tests, we determined NT-proBNP levels (measured using the PATHFAST Analyzer^TM^, with normal NT-proBNP values <300 pg/mL). Ruling in HF in the ED, age-adjusted cut-points for NT-proBNP were established: ≥450 pg/mL for patients under 50 years, ≥900 pg/mL for patients aged 50–75 years, and ≥1800 pg/mL for patients over 75 years. An estimated GFR (eGFR) was calculated from serum creatinine using the isotope dilution mass spectrometry (IDMS) traceable equation and the Chronic Kidney Disease Epidemiology Collaboration (CKD-EPI) equation.

Using the Simpson method, the left ventricular EF (LVEF) was calculated and measured by echocardiography.

The hospital’s ethics committee approved the study, and informed consent was obtained from all patients.

Statistical analysis was performed using IBM-SPSS version 25.0 for Windows. For continuous variables, the data are presented as numbers and percentages, median and interquartile range. The Pearson or Mantel–Haenszel chi-square test were used to demonstrate the association between variables. For comparison between the three groups of NT-proBNP levels of patients we used Kruskal–Wallis non-parametric test, with a *p*-value of <0.05 as statistical significance. ROC curves were constructed to assess whether the NT-proBNP levels can be used as a diagnostic test for heart failure. The null hypothesis was rejected at a threshold of 5% (*p* < 0.05). Linear regression analysis was performed to investigate the possible association between the NT-proBNP level and the variables correlated in the univariate analysis.

## 3. Results

A total of 302 patients were analyzed, out of which 182 were men (60.3%)—group A, n = 31 (17.03%), group B, n = 79 (43.4%), group C, n = 72 (39.57%)—without statistically significant differences regarding gender distribution between groups (*p* = 0.472, 95% CI, chi-square test). The clinical and paraclinical characteristics of the three groups are presented in Table 1. 

At hospital admission, most patients were in NYHA class III (n = 145, 48.01%) or IV (n = 154, 50.99%), with the majority of NYHA III in group B (56.1%, *p* < 0.001) and NYHA IV in group C (61.9%, *p* < 0.001) (Table 1).

Regarding the modifiable cardiovascular risk factors, most patients were non-smokers (n = 240, 79.4%), without significant differences between the three groups (*p* = 0.411). Alcohol consumption was higher in groups B and C, but without a statistically significant difference (*p* = 0.926). Patients in group C had lower cholesterol levels (median—131.00, interquartile range—[114.00–158.00] mg/dL) than those in group A (median—151.00, IQR—[156.00–178.00] mg/dL) and group B (median—147.00, IQR—[121.00–187.00] mg/dL), *p* = 0.005. Patients in group C were significantly older than those in groups A and B (74 years vs. 63 years and 70 years, respectively, *p* < 0.001), and had a lower weight at admission (72 [62.00–85.00] kg vs. 92 [79.75–109.25] kg and 80.5 [74.75–95.25], *p* < 0.001). 

Glycemia at admission analysis did not show any statistically significant differences (*p* = 0.957). The prevalence of diabetes was similar between the three groups (group A—22 (13.58), group B—72 (44.44), group C—68 (41.98), *p* = 0.67). 

The mean duration of hospitalization was longer in group C, 7 [5–9.75] days, compared to group A (5 [4–7] days) and group B (6 [5–8] days), but without reaching statistical significance (*p* = 0.19). 

The number of previous readmissions in group C was significantly higher than in groups B and A (1.00 [0–2.00] vs. 0 [0–1.00] vs. 0 [0–1.00], *p* = 0.01).

No significant differences were found between groups regarding heart rate (*p* = 0.681, Pearson χ^2^ = 0.768), systolic blood pressure (*p* = 0.182), or the presence of bundle branch block (*p* = 0.108, Pearson χ^2^ = 4.449). However, patients in group A presented a higher diastolic blood pressure at admission compared to groups B and C (*p* = 0.029). None of the patients received implantable pacemaker/cardioverter-defibrillator therapy during this study. 

No notable differences were found with respect to serum sodium (*p* = 0.213), potassium (*p* = 0.815), and chloride concentrations (*p* = 0.764). 

Concerning the etiology of heart failure, we found a higher prevalence of hypertension in groups B and C (*p* = 0.01). Tricuspid regurgitation was found to be more prevalent in patients with an NT-proBNP > 10,000 pg/mL (48.4%—group C vs. 44.6%—group B vs. 41.3%—group A, *p* = 0.018). No significant differences were found in respect to the prevalence of mitral regurgitation (*p* = 0.13), aortic stenosis (*p* = 0.76), or aortic regurgitation (*p* = 0.79). With regard to dilated cardiomyopathy, the majority had an ischemic etiology (diagnosed by coronary angiography), but without significant differences between the three groups (group A—n = 17 [63.7%] vs. group B—n = 39 [64.1%] vs. group C—n = 38 [64.4%], *p* = 0.34) (Table 2).

Patients with higher levels of NT-proBNP also expressed elevated levels of serum lactate (1.20 mmol/L, 95% CI [0.70–3.70] in group A vs. 1.75 mmol/L, 95% CI [0.80–4.20] in group B vs. 1.90 mmol/L, 95% CI [0.00–7.60] in group C, *p* = 0.016). 

Group C had higher serum creatinine levels, 1.24 [0.96–1.65], with lower glomerular filtration rates (eGFR): 52.00 [34.25–69.00] mL/min/1.73 m^2^, compared to those in group A (serum creatinine 0.90 [0.745–1.115], eGFR 81.00 [61.00–100.00] mL/min/1.73 m^2^) and group B (serum creatinine 0.90 [0.745–1.12] mg/dL, eGFR—61.00 [44.00–79.00] mL/min/1.73 m^2^), *p* < 0.001. Additionally, patients from group C had a higher degree of liver damage that was documented by increased levels of GPT transaminase (25.00 [14.00–55.00] IU/L), compared to group A (22.00 [13.00–62.50] IU/L) and group B (21.00 [14.25–48.00] IU/L), but without reaching statistical significance (*p* = 0.49). LVEF was significantly lower in group C (30.00% [20.00–40.00]) compared to group A (35.00% [29.50–40.00]) and B (34.00% [26.00–42.75]), *p* = 0.035.

The hemoglobin concentration was found to be lower in group C compared to groups A and B, reaching statistical significance (group C—12.85 [11.30–14.40], group A—13.60 [12.90–14.80], group B—13.25 [12.00–14.48], *p* = 0.028). 

The red cell distribution width analysis between the three groups showed that patients with higher levels of NT-proBNP express a higher RDW: group C—15.50% [13.95–16.85] vs. group A—14.15% [13.00–14.90] vs. group B—14.50% [13.50–16.00], *p* < 0.001. 

On linear regression analysis, the NT-proBNP levels correlate with admission weight and serum creatinine levels (*p* < 0.001), but with an R^2^ value of 0.3, well below the expected value of 0.8–1, showing that NT-proBNP levels may also depend on other uninvestigated factors. The median value of NT-proBNP is 6960 pg/mL [640–35,000] in patients only with HF, higher in those with HF and chronic kidney disease—9013 pg/mL [1148–30,000], but without a statistically significant difference (*p* = 0.43). The median NT-proBNP value is significantly lower in those with NYHA class III on admission (6569 pg/mL, [640–34,545]), compared to NYHA IV (10,090.5 pg/mL, [1004–35,000], *p* = 0.004).

In contrast, a significant association with NT-proBNP level was detected between the occurrence of death (*p* = 0.030, Pearson χ^2^ = 7.014), the type of diagnosis (HF ± renal impairment) (*p* = 0.039, Pearson χ^2^ = 6.465), and the NYHA class at hospitalization (*p* = 0.0027, Pearson χ^2^ = 11.001).

Patients in group C presented a 2.53-fold increase in the risk of kidney damage than those in group A (OR = 2.53, (1.22–5.27, 95% CI), *p* = 0.014, suggesting that NT-proBNP can serve as an adverse prognostic factor. 

In group C, more in-hospital cardiovascular deaths were identified, 17 pts (13.5%) compared with group A, 1 pt (2.2%), and group B, 6 pts (4.6), *p* < 0.001. Additionally, we found an association between the mean survival time and the NT-proBNP level (*p* = 0.045). 

**Figure 2:**
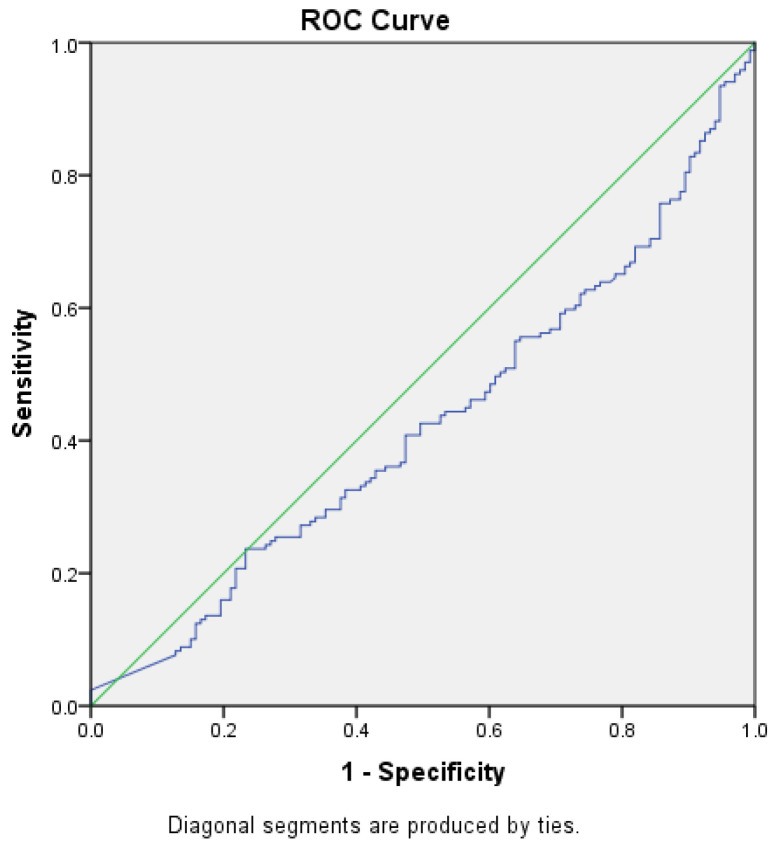
ROC curve (blue) for NT-proBNP levels as a predictor for HF without CKD (AUC = 0.426) Green—random classifier curve.

**Figure 3:**
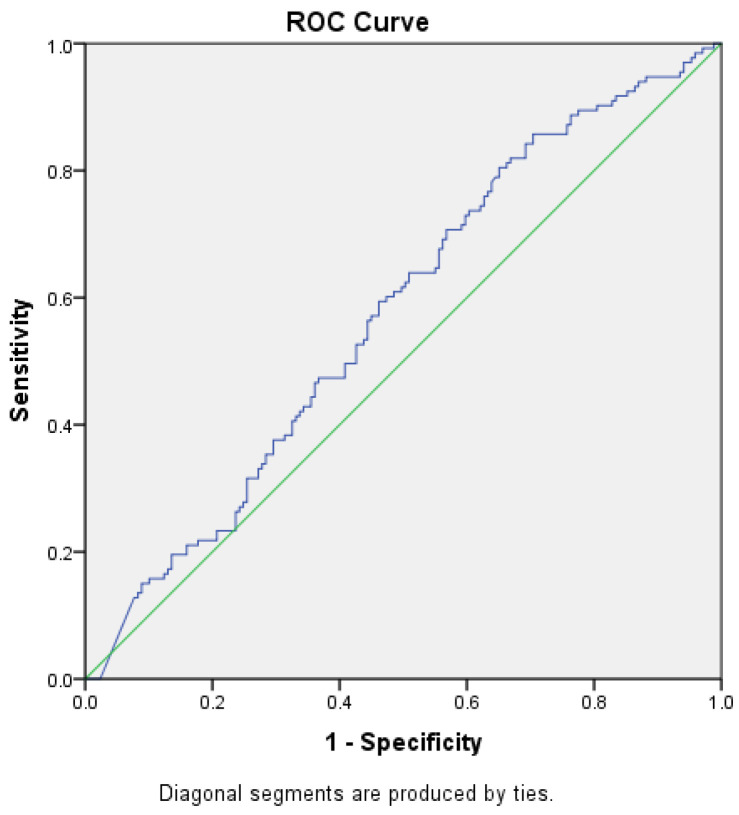
ROC curve (blue) for NT-proBNP levels as a predictor for HF with CKD (AUC = 0.574). Green—random classifier curve.

ROC curves were constructed to determine the best cut-off value for NT-proBNP level in prediction of heart failure, with or without renal impairment and to assess whether this analysis can be used for diagnostic purposes by obtaining a sensibility and specificity of the test closer to 100%, starting from the null hypothesis that the value for NT-proBNP level cannot make the difference between the patients with HF, with or without renal impairment, and those without the disease (Figure 2 and Figure 3).

The area under the ROC curve for the level of NT-proBNP and the diagnosis of HF without renal impairment was 0.426 (*p* = 0.026, 95% CI [0.361–0.49]), and for NT-proBNP level and renal impairment was 0.574 (*p* = 0.026, 95% CI [0.511–0.639]), reaching the statistical significance threshold in both situations. Thus, we can conclude that there might be an association between HF with or without concomitant renal impairment and NT-proBNP levels, but we could not demonstrate that this test, NT-proBNP level, can be used as a diagnostic test to predict HF (AUROC values closer to 0.5 than 1, the expected value) and no cut-off point could be established for the NT-proBNP level.

## 4. Discussion

The concentrations of NT-proBNP may vary widely according to the patient profile, the different clinical scenarios, and across the whole spectrum of EF—reduced, mildly reduced, and preserved. Therefore, their values should be interpreted accordingly to ensure a correct diagnosis [11]. Decompensated HF patients presenting to the ED with very high NT-proBNP concentrations, particularly those above 10,000 pg/mL, have a poor prognosis. They require hospital admission, usually in critical care, urgent investigation and close monitoring [18,19,20]. Additionally, based on the recommendations from the paper published in 2023 in the European Journal of Heart Failure [11], it is recommended for patients over 75 years of age to use an NT-proBNP level ≥ 1800 pg/mL for a positive diagnosis of heart failure. Because of these already existing data, we divided the patients in three groups: group A with NT-proBNP level < 3000 pg/mL, group B—NT-proBNP level between 3000–10,000 pg/mL, and group C—NT-proBNP level > 10,000 pg/mL). In our study, all patients in group C with NT-proBNP level > 10,000 pg/mL had a NYHA class III or IV on admission. This finding was also described in a study showing a 40% prevalence of patients with NT-proBNP level > 10,000 pg/mL on outpatients presenting with HF NYHA III or IV [21].

We found that the elevated NT-proBNP level (>10,000 pg/mL) correlates with the serum creatinine values and the weight at admission but without reaching the expected correlation significance values. The lower weight at admission in group C might be explained by the heart failure-induced sarcopenia. The patients with NT-proBNP levels > 10,000 pg/mL were significantly older compared to groups A and B. These findings correlate with the studies published by Kokkindis et al. and Chandrashekhar et al., which found that older patients with heart failure have an increased risk of sarcopenia induced by heart failure, and a subsequent decrease in body weight at admission [22,23]. There was no correlation between gender, smoking status, heart rate, presence of bundle branch block, and NT-proBNP levels above 10,000 pg/mL in all three groups (Table 1). However, the NT-proBNP levels were associated with NYHA class at hospitalization, the degree of concomitant renal impairment, and the occurrence of in-hospital death. Patients in group C with extremely elevated NT-proBNP have a higher risk of concomitant kidney damage, with a shorter survival time Moreover, LVEF was significantly lower in group C compared to groups A and B. In another study, Guglin et al. showed that there was no correlation between age, gender, and high NT-proBNP values (>4000 pg/mL, *r* = 0.29), but with a significant correlation with serum creatinine values (*p* < 0.0001). The authors concluded that renal dysfunction would be solely responsible for high NT-proBNP values, independent of HF severity [24]. 

We also found that patients from group C had a higher RDW, reaching statistical significance These findings are supported by Xanthopoulous et al. who suggested a correlation between a higher RDW and disease severity, NT-proBNP levels, left ventricular end-diastolic pressure, and left ventricular deformity [25,26].

Additionally, patients with higher levels of NT-proBNP also expressed higher serum lactate levels. Based on the study published by Uyar et al., a higher lactate level at admission is associated with higher hospitalization rates for heart failure at six months and an increased cardiovascular mortality [27].

In patients with HF, elevated levels of NT-proBNP are an independent factor that correlates with in-hospital mortality, number of previous readmissions, and cardiovascular mortality [28,29]. Furthermore, high NT-proBNP levels on admission are predictors of unfavorable outcomes [30], which was also demonstrated in our study. Bózsik B et al. described a correlation between NT-proBNP levels > 10,000 and in-hospital mortality, without finding an apparent cause to explain the high NT-proBNP values on admission [31]. The same was also described by Law C et al. in a paper that concluded that high NT-proBNP values (>3000 pg/mL) are not an indicator of HF severity, with more than a quarter of patients with high values having no signs of decompensated HF [32]. In our study, we found that high NT-proBNP values (above 10,000 pg/mL) correlate with the presence of HF and concomitant renal impairment, regardless of age. These data were like those of a recent study by Cui H. et al. which included only the elderly, suggesting that elevated NT-proBNP levels correlate with renal dysfunction in patients over 80 years of age and can be considered a marker of severity [33].

In the study by Tsuji H. et al., a correlation was described between low serum hemoglobin and NT-proBNP levels, independent of age, gender, serum creatinine level, LV wall abnormalities, LV hypertrophy, or other cardiovascular risk factors [34]. This correlation was also found in our study.

## 5. Conclusions

Patients hospitalized with decompensated HF and very high levels of NT-proBNP, above 10,000 pg/mL at admission, are older, have a lower LVEF, higher NYHA class, a higher incidence of renal dysfunction, more extended hospital stay, a higher readmission rate for decompensated HF, with a shorter survival time resulting in a severe clinical profile.

The presence of very high levels of NT-proBNP may identify a special category of patients with a more severe prognosis that requires immediate hospitalization, more aggressive in-hospital management, and a closer follow-up after hospital discharge.

## Figures and Tables

**Table 1:** The clinical and paraclinical characteristics of the patients enrolled in this study (n = 302) according to NT-proBNP levels (Group A (n = 46, NT-proBNP level < 3000 pg/mL), Group B (n = 130, NT-proBNP level between 3000–10,000 pg/mL), and Group C (n = 126, NT-proBNP level > 10,000 pg/mL)).

Characteristics	Total (n = 302)	Group A (n = 46)	Group B (n = 130)	Group C (n = 126)	*p*-Value
Age (y)	70 [61–79]	63 [52.50–69.00]	70 [61.00–78.00]	74 [66.00–81.25]	*p* < 0.001
Gender (females), n (%)	120 (39.7)	15 (12.5)	51 (42.5)	54 (45.00)	*p* = 0.472
Gender (males), n (%)	182 (60.3)	31 (17.03)	79 (43.40)	72 (39.57)	*p* = 0.472
Hospitalization (days)	7 [5–8]	5 [4–7]	6 [5–8]	7 [5–9.75]	*p* = 0.19
No. of readmissions (n)	0 [0–1.00]	0 [0–1.00]	0 [0–1.00]	1.00 [0–2.00]	*p* = 0.01
Deaths (in-hospital)	41 (13.5)	2 (4.3)	15 (11.5)	24 (19.04)	*p* = 0.0172
LVEF (%)	30.00 [25.00–40.00]	35.00 [29.50–40.00]	34.00 [26.00–42.75]	30.00 [20.00–40.00]	*p* = 0.035
Smoking, n (%)	91 (30.13)	17 (18.70)	43 (47.25)	31 (34.06)	*p* = 0.411
Alcohol, n (%)	47 (15.56)	8 (17.03)	22 (46.80)	17 (36.17)	*p* = 0.926
SBP at admission (mmHg)	140 [120–150]	140 [130–158.50]	140 [120–150]	135 [120–157.50]	*p* = 0.182
DBP at admission (mmHg)	80 [75–90]	90 [80–95]	80 [70–90]	80 [70.5–90]	*p* = 0.029
HR at admission (b/min)	96 [80–114.50]	91.5 [80–115]	93 [80–110]	100 [80–120]	*p* = 0.56
Weight at admission (kg)	80 [69.45–94.00]	92 [79.75–109.25]	80.5 [74.75–95.25]	72 [62.00–85.00]	*p* ≤ 0.001
Glycemia (mg/dL)	124 [105.25–162.75]	121 [101.00–170.00]	124 [106.25–161.75]	125 [106.00–162.00]	*p* = 0.957
Diabetes, n (%)	162 (53.64)	22 (13.58)	72 (44.44)	68 (41.98)	*p* = 0.67
Afib, n (%)	154 [51.00]	20 (13.00)	71 (46.10)	63 (40.90)	*p* = 0.412
QRS duration (ms)	111 [98.00–131.00]	112 [98–128]	112 [98–130]	110 [97–136]	*p* = 0.937
LBBB (%)	86 (28.47)	8 (09.30)	38 (44.19)	40 (46.51)	*p* = 0.176
Hb (g/dL)	13.20 [12.00–14.50]	13.60 [12.90–14.80]	13.25 [12.00–14.48]	12.85 [11.30–14.40]	*p* = 0.028
RDW (%)	14.70 [13.60–16.20]	14.15 [13.00–14.90]	14.50 [13.50–16.00]	15.50 [13.95–16.85]	*p* < 0.001
Serum creatinine (mg/dL)	1.12 [0.90–1.45]	0.90 [0.75–1.12]	1.12 [0.92–1.42]	1.24 [0.96–1.65]	*p* < 0.001
eGFR (mL/min/1.73 m^2^)	60.00 [43.00–79.00]	81.00 [61.00–100.00]	61.00 [44.00–79.00]	52.00 [34.25–69.00]	*p* < 0.001
Total cholesterol (mg/dL)	140.00 [119.00–142.00]	151.00 [126.00–178.00]	147.00 [121.00–187.00]	131.00 [114.00–158.00]	*p* = 0.005
Na^+^ (mmol/L)	138.00 [136.00–140.00]	139.00 [137.25–140.75]	138.00 [135.00–140.00]	138.00 [135.00–141.00]	*p* = 0.213
K^+^ (mmol/L)	4.40 [4.00–4.775]	4.40 [4.00–4.80]	4.40 [4.00–4.60]	4.40 [4.00–4.90]	*p* = 0.815
Cl^−^ (mmol/L)	102.00 [97.00–105.00]	101.00 [96.75–105.00]	101.00 [96.25–104.75]	102.00 [98.00–105.00]	*p* = 0.764
Lactate (mmol/L)	1.80 [1.30–2.60]	1.20 [1.10–1.60]	1.75 [1.50–2.325]	1.90 [1.30–3.05]	*p* = 0.016
NT-proBNP (pg/mL)	8240.00 [4130.00–15,003.50]	1988.50 [1585.50–2607.50]	5574.50 [4231.50–7511.75]	17,554.00 [12,890.25–28,399.25]	*p* < 0.001
GPT (UI/L)	24.00 [14.00–53.75]	22.00 [13.00–62.50]	21.00 [14.25–48.00]	25.00 [14.00–55.00]	*p* = 0.49
NYHA I, n (%)	0 (0)	0 (0.0)	0 (0.0)	0 (0.0)	
NYHA II, n (%)	3 (1.00)	1 (2.2)	1 (0.8)	1 (0.8)	
NYHA III, n (%)	145 (48.01)	25 (54.3)	73 (56.1)	47 (37.3)	
NYHA IV, n (%)	154 (50.99)	20 (43.5)	56 (43.1)	78 (61.9)	

n = number of patients. *p*-value = statistical significance level (comparison between group A vs. group C). CI = confidence interval. LVEF—left ventricular ejection fraction, SBP—systolic blood pressure, DBP—diastolic blood pressure, HR—heart rate, AFib—atrial fibrillation, LBBB—left bundle branch block, Hb—hemoglobin, K^+^—serum potassium, eGFR—estimated glomerular filtration rate, GPT—glutamate-pyruvate transaminase.

**Table 2:** Causes of heart failure between the three groups. There is an association between tricuspid regurgitation, mitral regurgitation, dilative cardiomyopathy, and hypertension with elevated levels of NT-proBNP (comparison between group A vs. group C).

Etiology	Group A	Group B	Group C	*p*-Value
Hypertension	28 (60.9)	71 (54.6)	60 (47.6)	0.01
Dilative cardiomyopathy (ischemic and non-ischemic)	27 (58.7)	61 (46.9)	59 (46.8)	0.013
Mitral regurgitation	17 (37.0)	67 (51.5)	82 (65.1)	0.13
Mitral stenosis	0 (0.0)	4 (3.1)	3 (2.4)	0.98
Aortic regurgitation	1 (2.2)	4 (3.1)	11 (8.7)	0.79
Aortic stenosis	1 (2.2)	7 (5.4)	19 (15.1)	0.76
Tricuspid regurgitation	19 (41.3)	58 (44.6)	61 (48.4)	0.018

## Data Availability

All data and materials supporting the present study’s results are available upon request from the corresponding author. The data are not publicly available to limit the amount of publicly available personal information, as classified by the European Union General Data Protection Regulation.
